# Improving patients’, carers’ and primary care healthcare professionals’ experiences of discharge communication from specialist palliative care to community settings: a protocol for a qualitative interview study

**DOI:** 10.1186/s12904-024-01451-1

**Published:** 2024-06-20

**Authors:** Katharine Weetman, John I. MacArtney, Catherine Grimley, Cara Bailey, Jeremy Dale

**Affiliations:** 1 Institute of Clinical Sciences, Birmingham Medical School, University of Birmingham, Birmingham, B15 2TT UK; 2 Unit of Academic Primary Care, Warwick Medical School, University of Warwick, Coventry, UK; 3 Marie Curie Hospice West Midlands, Solihull, West Midlands UK

**Keywords:** Palliative care, Hospice care, Patient discharge summaries, Transitional care, Communication

## Abstract

**Background:**

Patients who have benefited from specialist intervention during periods of acute/complex palliative care needs often transition from specialist-to-primary care once such needs have been controlled. Effective communication between services is central to co-ordination of care to avoid the potential consequences of unmet needs, fragmented care, and poor patient and family experience. Discharge communications are a key component of care transitions. However, little is known about the experiences of those primarily receiving these communications, to include patients’, carers’ and primary care healthcare professionals. This study aims to have a better understanding of how the discharge communications from specialist palliative care services to primary care are experienced by patients, carers, and healthcare professionals, and how these communications might be improved to support effective patient-centred care.

**Methods:**

This is a 15-month qualitative study. We will interview 30 adult patients and carers and 15 healthcare professionals (*n* = 45). We will seek a range of experiences of discharge communication by using a maximum variation approach to sampling, including purposively recruiting people from a range of demographic backgrounds from 4–6 specialist palliative care services (hospitals and hospices) as well as 5–7 general practices. Interview data will be analysed using a reflexive thematic approach and will involve input from the research and advisory team. Working with clinicians, commissioners, and PPI representatives we will co-produce a list of recommendations for discharge communication from specialist palliative care.

**Discussion:**

Data collection may be limited by the need to be sensitive to participants’ wellbeing needs. Study findings will be shared through academic publications and presentations. We will draft principles for how specialist palliative care clinicians can best communicate discharge with patients, carers, and primary care clinicians. These will be shared with clinicians, policy makers, commissioners, and PPI representatives and key stakeholders and organisations (e.g. Hospice UK) and on social media. Key outputs will be recommendations for a specialist palliative care discharge proforma.

**Trial registration:**

Registered in ISRCTN Registry on 29.12.2023 ref: ISRCTN18098027.

**Supplementary Information:**

The online version contains supplementary material available at 10.1186/s12904-024-01451-1.

## Introduction

### Background

Palliative care provides care and support for people with terminal illnesses and those at the end of their life who are dying, as well as for their close persons. In the UK, it is often called generalist palliative care when provided by health and social care professionals in hospitals or the community, and primary palliative care when provided by healthcare professionals in primary care e.g. community nurses and general practitioners (GPs) [1]. Those with acute or complex palliative care needs can be referred to *specialist* palliative care (e.g. hospices), which is not only for people in the last days of life, but is available to intervene and support people with life-limiting illnesses whenever they develop complex palliative needs [2–4]. Patients who have benefited from specialist intervention during periods of acute/complex palliative care needs often transition from specialist-to-primary care where their day-to-day healthcare needs will be managed by general practice and community teams, once such needs have been managed and/or controlled [5]. A previous systematic review on hospice discharge [6] estimated discharge rates from 5–23%.

Effective communication between services during care transitions known as “discharge communication” is central to co-ordination of care to avoid the potential consequences of unmet needs, fragmented care, and poor patient and family experience. Miscommunications and unclear information can result in a lack of patient-centred care [7] and continuity of care [8], confusion and anxiety [9], and avoidable crises such as readmission as an emergency [10]; such readmissions may be unnecessary and/or preventable as they could be avoided or at least reduced with better co-ordinated care transitions [11] and improved communication and information continuity/sharing [12]. However, if communication is effective and involves patients in a way that respects their choices and needs, this can lead to benefits such as improved well-being [9], increased satisfaction [13], and better understanding of how to manage their symptoms [14, 15]. Indeed, it has been good practice for over 20 years in the UK for patients to receive copies of written communications sent between their physicians [16–19].

Our recent study looking at hospice discharge in five UK hospices, indicated that hospice patients (and where relevant, their carers) are not consistently receiving or being offered discharge letters [5]. Although hospice care seeks to provide a holistic service, we also found that there was a focus on physical needs in these letters, with much less focus on psychological/emotional and social needs, with spiritual needs being rarely documented (2.4%) [5].

Our previous research [5, 20] found heterogeneity in the quality of specialist palliative care discharge communications to primary care, along with the inconsistencies in copying in patients to discharge communication. We also found that little is known about how being discharged from specialist palliative services affects patients’ and carers’ experiences of end of life care, or what information community teams need for managing the patient’s ongoing care;

Improving discharge communication has the potential to increase shared understanding of the patient’s condition, their symptoms and planned management of pain, symptoms and holistic needs. Improved communication should empower those receiving such information to better enact the patient’s chosen advance care plan [5, 20], which may in turn improve a patient’s quality of life and experience of death and dying. Therefore, this study aims to understand how discharge communications from specialist palliative care services to primary care are currently being experienced by those receiving them, and how this can be improved in order to ensure improved care at this crucial and time-sensitive part of the healthcare journey.

### Research question

How can specialist palliative care discharge communications to primary care better support patient and carer needs?

## Methods

### Objectives

1. Explore patient and carer experiences of discharge communication from specialist palliative care to identify how it currently supports their needs and how it might be improved.
2. Investigate how primary healthcare professionals currently experience specialist palliative care discharge communication and how it might be improved to support joined-up care for people with palliative needs.
3. Synthesise findings to inform recommendations about how discharge communication from specialist palliative care services can be improved.

### Design

This is a qualitative study exploring the lived experiences of those receiving specialist palliative care discharge communications. Qualitative methods [21] are well suited for generating rich data drawing on participants’ accounts, and will allow us to explore experiences in relation to their contextual settings.

### Theoretical framework

This qualitative research is positioned within an interpretative paradigm [22]. Crucial to our approach are reflexive practices, the ways in which both ours and our participants’ bounded and partial positions become knowable [23, 24]. A qualitative approach is appropriate for the proposed research as we engage participants about their experiences and explore the meanings and interpretations of the discharge communication event(s).

### Setting

General practices and specialist palliative care services provided by hospices and hospitals in the West Midlands. The West Midlands in England, United Kingdom (UK), has the largest ethnically diverse population outside of London distributed across a range of geographical locations, from inner city to rural areas [25]. This is a multicentre project within this geographical region. Participating sites will be sampled for deliberate heterogeneity of sociodemographic characteristics of the public population for which the site cares for and seeks to serve e.g. indices of deprivation, urban or rural setting, patient mean age group and ethnicity. Therefore, there will be diversity and variation of practice locality.

### Participants

Adult patients and carers who have had recent experience of discharge from specialist palliative care. Primary healthcare professionals including general practice team members and district nurses.

### Sampling

To ensure the findings have sufficient depth and information power [26], we will purposefully recruit [27] from 4–6 specialist palliative care services located at either hospice or hospital sites, and 5–7 general practices to provide participant diversity.

We seek to collect 30 recent discharge experiences of patients and /or carers. Should ongoing community support be being received, participants will still be eligible to take part. Where interviews take place with patients and carers in dyads, this will be counted as a single “experience”, such that one person’s account cannot be disaggregated from the others. The sampling timeframe was discussed and endorsed by our PPI, who felt people should be spoken to as soon as possible after discharge for memory recall, ensuring relevance, and respecting that participants may not have long left to live.

We will also interview 15 primary care health professionals with experience of receiving patients discharged from specialist palliative care. We will recruit for healthcare professional diversity in regards to role (general practitioners, district and practice nurses…), setting and locality, specialty/special interest areas, and grade/experience.

The inclusion and exclusion criteria for the selection and screening of all participants is found in Table 1 below.

**Table 1:** study inclusion and exclusion criteria for screening and selection of participants

Inclusion criteria	Exclusion criteria (all)
*For patients and carers only:* • Adult (18 + years) patients or carers^a^ for patients discharged^b^ to primary care from a participating specialist palliative care facility^c^ at a hospital or hospice following an episode of care • Discharge event no longer than two weeks ago at the time of participant screening and selection for the study *For primary care healthcare professionals only:* • Working in a participating general practice team (including associated community nursing) and receive and/or act upon discharge communications. This may include, but not be limited to, general practitioners, nurse practitioners, and district nurses	Children (those aged < 18 years old) • Patients who are deemed by their care team or treating clinician as lacking capacity to provide informed consent to participate in the study (e.g. Alzheimer’s) or are otherwise deemed unsuitable for study participation • Patients discharged to providers or units other than primary care (e.g. discharge to secondary care) • Persons who have expressed a prior wish not to participate in research • Bereaved carers (< 6 months) • Healthcare professional who has worked for less than one month in Primary Care

^a^For the purposes of this study, we define a carer as the primary carer or any person (e.g. friend, relative…) who provides support for daily living activities (e.g. cooking) or personal care (e.g. washing) but is not directly employed to do so

^b^For the purposes of this study, we define discharge as any episode of inpatient or outpatient specialist palliative care (complete or incomplete)

^c^Not all hospital trusts have palliative care wards as a separate designated service. Therefore, discharges from other specialties (e.g. Gastroenterology) will be eligible so long as the patient has also been under the care and support of the specialist palliative care facility/staff member at that participating site

The total sample size of *n* = 45 has been devised using the principles of the ‘information power matrix’ [26]. The matrix helps us anticipate the pressures for ‘more’ or ‘less’ interviews and provides a balance between fulfilling: (a) the broad explanatory aim of this study – there has been very little research into experiences of discharge from specialist palliative care (*more*); (b) a need to explore several specific locales (hospices, hospitals, and general practices) and demographic groups (gender; socio-economic status; ethnicity; sexuality; disability) (*more*); (c) the specific and applied contribution this study provides to the established theories and literature on discharge communication that exists in other healthcare fields (*less*); (d) the in-depth quality of dialogue we expect to collect – accounts of discharge are expected to be detailed and contain experiences of ‘before’ and 'after’ (less); and, (e) the thematic focus of our analytical strategy (less).

### Equality and diversity

This research, as far as possible will ensure and promote equality and diversity from design, through to implementation, delivery and dissemination. The study will collect data on age, sex, sexuality, religion, disability, and ethnicity. The study will be introduced to eligible participants as they are being discharged from specialist palliative care to mitigate the barriers that may be experienced from mail out invitations alone. Professional translators will be provided if required to ensure that participants can take part in their preferred language and that this is not a barrier to participation.

### Recruitment

To ensure patient confidentiality, participating hospices and hospitals will be responsible for screening, identifying, and inviting eligible patient and carer participants for the study. The assessment of capacity will be undertaken by the patient’s clinical team at the site.

Eligible participants will be signposted to the study verbally and provided with an electronic or hard copy invitation. The study invitation will include a copy of the invitation letter, participant information sheet, and consent form. Those eligible may also be contacted retrospectively by the direct care team, and/or provided a reminder after 48 h – this is in acknowledgement that palliative care discharges can be rapid and/or unplanned in practice [28]. Interested participants will be given a minimum of 24 h to decide if they wish to take part and may directly contact the research team with questions and to agree a time, date, and place for the interview.

All eligible staff at participating primary care sites will be invited to take part in interviews, by the staff member/route of contact for site recruitment or an appropriate colleague (e.g. CRN nurse or GP champion). This study invitation will be electronic and/or hard copy and include a copy of the invitation letter, participant information sheet, and consent form.

### Data collection and interview procedure

Interviews will be semi-structured and will seek to elicit the participants’ views on discharge from specialist palliative care, any discharge communications or discharge letters they were party to, and when these were received, as well as their views on what currently works (or not), and their suggestions for improvements to discharge communications. The interview schedule (Supplementary files 1 and 2) are informed by our previous research involving interviews on discharge communications [9, 29].

Each participant will only take part in one interview with the research team, which is expected to take no more than one hour. However, interview timing will be adjusted flexibly to meet the needs and preferences of participants.

Patient and carer: Participants will be offered interviews via online video (e.g. Microsoft Teams or Zoom), phone, or in-person at the patient or carer’s home, hospice, or another place of their choice. Given the potential vulnerability of the participant population, infection control will be followed for in-person contact to include encouraging covid-19 lateral flow testing prior and wearing of face masks; any other reasonable requests to reduce the risk of viral infection will be respected and adhered to wherever practicably possible.

Healthcare professionals: Interviews will be flexible and more rapid in recognition of the pressures on general practice (10–30 min); structured topic guides will be used to ensure minimal disruption. Interview dyads or groups (≤ 3) will be offered, whereby persons can be interviewed concurrently/in groups. This method has closer likeness to that of an individual interview, as opposed to a focus group [7].

All interviews will be recorded and transcribed. Demographic information on participants will be collected at interviews to monitor the purposive sampling objectives. Appropriate steps will be taken to ensure participant confidentiality and interview transcripts will be pseudonymised to include the removal of any direct identifiers.

### Consent process

Informed consent for this study for all participants will be sought both verbally and recorded in written form using the study consent form (supplementary file 3). Before each interview commences, the researcher will review the study materials with the participant, which will have been provided in advance, and invite any questions. The researcher will confirm with the participant their understanding of the study. In all cases, the consent form will be co-signed by the interviewer. The study materials clearly state that participation is voluntary and participants can decline or withdraw without reason or consequence and choosing not to take part will not affect participant medical or legal rights in any way.

### Inclusion and accessibility

Participants will be asked when arranging the interview, what their accessibility needs are and how best the research team can accommodate these. The consent process has been deliberately designed to maximise inclusivity and accessibility. This is because some of the participants for this study will likely have life-limiting conditions which can affect mobility and small motor skills, such as Motor Neurone Disease or following chemotherapy, radiation, or stroke. Members of the research team have lived experience of disability and palliative care (personally and/or professionally). Efforts to limit unintended exclusion from the study include the following. First, the consent form can be completed in hard copy using a wet ink signature and/or in electronic form. Combination completion will also be permitted as suited to participant preference. For electronic completion, the boxes in the word document consent form have been formatted so that clicking in the large square box enters a "tick". The electronic signature in the end declaration can then be electronically signed and/or typed. The reasons for tick box consent for each section are to support participants who may struggle with initialling boxes. This is an adaption that has been made to the HRA template but is in alignment with HRA e-consent guidance (2019) [30]. Second, proxy signing has also been designed into this study to allow participation by persons who are able to read and process information, and provide informed consent, but may not have the physical ability to complete the written consent form (e.g. difficulty in coordination, mobility or writing). The research team acknowledge in some circumstances that electronic completion of the consent form may be difficult or prohibiting for those persons with disabilities or health conditions that affects their mobility or small motor skills. Therefore, a participant can nominate a proxy such as a carer or companion (e.g. friend, partner, family member), healthcare professional, or a member of the research team to tick the boxes and type/sign their name in the signature line for them. The use of proxy signing will be participant led to ensure autonomy and dignity is prioritised at all times. The consent form has been designed so that proxy signing will be clearly indicated and recorded in all cases that apply.

### Study participant support

It is a common occurrence that when interviewing people with serious illnesses or terminal conditions that they will often prefer to have a close person with them during the interview for support [31]. Study participants will therefore have the option to attend the interview alone or with a carer or close person. The carer/support person can listen only, or if they wish may be able to take part in the interview in which case a signed consent form will be required. Where patients and carers, relating to the same case, have both been invited to take part in the study, they can be interviewed together (joint interview or dyad) or separately.

If a participant becomes upset during an interview the researcher will remind the participant that they can have breaks or stop the interview at any time. The participant information sheet provides a list of supportive resources the participant can access and they will also be reminded that they can discuss their concerns with their doctor. No medical advice will be provided at interviews.

### Methods for sharing study findings with participants

The results of the study will be shared with all living study participants (unless they would prefer not to receive this) in a lay summary which will be co-produced with our PPI members (see below). If a study participant has died, the study findings can be shared with a nominated person, which will be ascertained as part of the consent process. Participants will also be signposted to any study webpages hosted by the University of Birmingham and University of Warwick, which will be updated as outputs are published and produced e.g. to read the open access peer-reviewed paper.

### Payments, rewards, and recognition for study participants

Patient and carers will be provided with a £25 high street shopping voucher as a thank you for participating. An acknowledgement of the contribution of study participants will be provided in outputs such as publications as a collective statement. Travel has also been costed as necessary. Clinical sites will be reimbursed for staff time and any other costs.

### Patient and public involvement

To develop the study proposal and assess the acceptability of the research, we consulted with an existing palliative care PPI group associated with BRHUmB – a NIHR funded Research Hub for Palliative and End of LIfe Care in the West Midlands. These PPI members reflect a diverse range of health, social, and cultural needs and have all experienced palliative care as a patient, carer or volunteer. We held an initial meeting with the BRHUmB PPI group and the group members recognised the issues around poor communication and how it can affect care following discharge and cause confusion.

Three PPI members with varying backgrounds and experience of palliative care have joined the research advisory team. One member was involved in developing the patient facing materials and ensuring readability and accessibility. The PPI group will advise on research design, recruitment, and where they wish to the analysis, writing and dissemination of findings. They will be invited to a total of 10 meetings during the 15-month study to include PPI meetings, advisory meetings with the research team, and a dissemination workshop.

### Analysis

Interview data will be analysed with reflexive thematic analysis [23, 24, 32, 33]. This will involve synthesis and interrogation of interview transcripts to identify themes in the data by CG and KW in line with the six stages of reflexive thematic analysis. We will use NVivo software to support the processes of (i) familiarisation, (ii) generating codes and (iii) constructing themes [23]. KW and CG will draw on existing relevant literature and discuss potential themes with JM, the research team, PPI representatives, and the advisory group as part of the iterative process of (iv) refining codes and (v) defining themes, throughout the ongoing interviews and during the process of (vi) writing-up [23].

### Development of recommendations

The final study advisory group will take the form of a hybrid half day collaboration workshop. In addition to clinical, academic and PPI group members, we will invite relevant stakeholders and collaborating commissioners to attend. We will share initial findings and pseudonymised excerpts from the interviews, using a Modified Nominal Group Technique [34] (M-NGT), a collaborative and consensus building approach, to generate transferrable insights to best inform and translate findings to practice and policy. We will also co-produce a list of recommendations for discharge communications from specialist palliative care.

## Discussion

This study is important because it will be one of the first to provide an in-depth consideration into how discharge communications from specialist palliative care might be improved to support effective patient-centred care at the end of life [5]. Our use of qualitative methods and inclusive approach to data collection will help to ensure that the experiences of people with life-limiting conditions are not “forgotten” and will help to ensure that palliative and primary care services are able to support their quality of life through provision of timely and holistic care [5, 35].

### Strengths

This study will produce evidence that can inform improvement in communication between specialist palliative services, general practice and community teams, and patients and their carers. Informed by our patient and public involvement work, and through listening to peoples’ experiences of discharge from specialist palliative care, this research will focus on making communication better for all involved. It will highlight the need to continue to prioritise discharge communications through evidencing deficits and providing empirically-based recommendations for practice [7]. Whilst there are specialised discharge templates for emergency care and mental health [36], they have not yet to our knowledge been developed for specialist palliative care in the same way. As an outcome of this work, we will develop a set of recommendations for palliative care discharge communications, designed to address the variable quality that currently exists. This research will bring improvements to patient and carer experiences as well as inform routes for better communication and integration of care between services.

### Limitations

There are several limitations to this study that arise from the need to ensure data is collected in an ethical and appropriate way. Research with people at the end of life, and those who support them, needs to take into account the personal, emotional and social difficulties people can experience during this time [5]. Potential participants will be identified by clinicians to ensure that those who are finding this time particularly difficult are not troubled. However, this may mean that those who are most in need of most discharge support are not afforded the opportunity to contribute to the study. We may also need to wait days or weeks to speak to participants, to ensure participation does not result in an undue emotional burden upon them. As the study is exploring participant’s accounts of their experiences of discharge, this delay may lead to poorer recall of events and feelings.

### Dissemination and impact strategy

Study findings will be shared through academic publications and presentations. Through our engagement and dissemination activities, we hope to raise awareness of patient’s entitlement to have a copy of their discharge letter. This study will also inform future research with the need to implement and evaluate the recommended principles to potentially generate a palliative care discharge proforma. We expect our findings will be submitted to the NHS England and PRSB (Professional Records Standards Body) for consideration to affect policies as providing quality care in community settings becomes increasingly important. Such policy recommendations are likely to have a relatively short timeframe to impact and be supported by professional bodies, Colleges, and organisations that have previously supported similar initiatives, such as the Royal College of Physicians and Academy of Medical Royal Colleges.

## Conclusion

This study will provide important insights into the experiences of being discharged from specialist palliative care to primary care that have, so far, been significantly underexplored. It will provide an in-depth evidence base from which to develop recommendations and principles of good discharge practice for palliative care. The findings and recommendations will be of relevance both across the UK, but also to those responsible for transferring care from specialist palliative care to primary care in healthcare services around the world.

### Supplementary Information

Supplementary Material 1.

Supplementary Material 2.

Supplementary Material 3.

## Data Availability

The datasets generated during and/or analysed during the current study will be available upon request in accordance with the below. The full datasets will not be made available due to the potentially identifiable nature of the in-depth qualitative data. Access to patient-identifiable data will be restricted to members of the study co-ordination team who require it for the performance of their role, inclusive of the research team. However, the data and results may be used for secondary analysis for future research and/or research involving modified or different research questions; this may be undertaken by the research team and other researchers. In the latter case, data will only be shared in secure and pseudonymised form with other researchers. Data will be stored in line with University policy for 10 years and then destroyed.

## Abbreviations

ACP: Advanced care plan
CRN: Clinical research network
GP: General practitioner
HRA: Health research authority
NHS: National health service (UK)
UK: United Kingdom

## References

[1] Murray SA, Firth A, Schneider N (2015). Promoting palliative care in the community: production of the primary palliative care toolkit by the European Association of Palliative Care Taskforce in primary palliative care. Palliat Med.

[2] Clark D (2007). From margins to centre: a review of the history of palliative care in cancer. Lancet Oncol.

[3] NIHR. Better endings: right care, right place, right time: an independent review of NIHR research on end of life care services. NIHR Dissemination Centre. 2015. Available from: https://evidence.nihr.ac.uk/wp-content/uploads/2020/03/Better-endings-FINAL-WEB.pdf. Accessed 21 Jul 2021.

[4] Yardley S (2018). Deconstructing complexity: finding opportunity in problems shared. Palliat Med.

[5] Weetman K, Dale J, Mitchell SJ (2022). Communication of palliative care needs in discharge letters from hospice providers to primary care: a multisite sequential explanatory mixed methods study. BMC Palliat Care.

[6] Wu S, Volker DL (2019). Live discharge from hospice: a systematic review. J Hosp Palliat Nurs.

[7] Weetman K. An investigation of written discharge communication between hospital clinicians, GPs, and patients in the UK [PhD thesis]. University of Warwick; 2020. http://webcat.warwick.ac.uk/record=b3714883~S15.

[8] Thelen M, Brearley SG, Walshe C. A grounded theory of interdependence between specialist and generalist palliative care teams across healthcare settings. Palliat Med. 0(0):02692163231195989. Available from: https://journals.sagepub.com/doi/abs/10.1177/02692163231195989. 10.1177/02692163231195989 37691459

[9] Weetman K, Dale J, Scott E (2020). Adult patient perspectives on receiving hospital discharge letters: a corpus analysis of patient interviews. BMC Health Serv Res.

[10] Karasouli E, Munday D, Bailey C (2016). Qualitative critical incident study of patients’ experiences leading to emergency hospital admission with advanced respiratory illness. BMJ Open.

[11] Zhang Y, Luth EA, Phongtankuel V, et al. Factors associated with preventable hospitalizations after hospice live discharge among Medicare patients with Alzheimer's disease and related dementias. J Am Geriatr Soc. Available from: https://agsjournals.onlinelibrary.wiley.com/doi/abs/10.1111/jgs.18505. 10.1111/jgs.18505 PMC10771532 37417691

[12] Turbow SD, Ali MK, Culler SD (2023). Association of fragmented readmissions and electronic information sharing with discharge destination among older adults. JAMA Netw Open.

[13] Baxter S, Farrell K, Brown C (2008). Where have all the copy letters gone? A review of current practice in professional-patient correspondence. Patient Educ Couns.

[14] Weetman K, Dale J, Scott E (2021). Discharge communication study: a realist evaluation of discharge communication experiences of patients, general practitioners and hospital practitioners, alongside a corresponding discharge letter sample. BMJ Open.

[15] Reilly MO, Cahill M, Perry IJ (2005). Writing to patients: ‘putting the patient in the picture’. Ir Med J.

[16] Department of Health. Copying letters to patients: good practice guidelines. 2003. Available from: https://webarchive.nationalarchives.gov.uk/20120504030618/. http://www.dh.gov.uk/prod_consum_dh/groups/dh_digitalassets/@dh/@en/documents/digitalasset/dh_4086054.pdf

[17] National Institute for Health and Care Excellence (NICE). Patient experience in adult NHS services: improving the experience of care for people using adult NHS services. Clinical guideline [CG138]. 2012. Available from: https://www.nice.org.uk/guidance/cg138. Accessed 25 Feb 2019. 31909930

[18] The Academy of Medical Royal Colleges. Please, write to me: writing outpatient clinic letters to patients. 2018. Available from: https://www.aomrc.org.uk/reports-guidance/please-write-to-me-writing-outpatient-clinic-letters-to-patients-guidance/. Accessed 10/06/20.

[19] Department of Health (2000). The NHS plan: a plan for investment a plan for reform.

[20] Finucane AM, Swenson C, MacArtney JI (2021). What makes palliative care needs complex? A multisite sequential explanatory mixed methods study of patients referred for specialist palliative care. BMC Palliat Care.

[21] Green J, Thorogood N (2014). Qualitative methodology and health research.

[22] Greene J (2007). Mixed methods in social inquiry.

[23] Braun V, Clarke V. Thematic analysis. In: Cooper H, Camic PM, Long DL, Panter AT, Rindskopf D, Sher KJ, editors. APA handbook of research methods in psychology. Research designs: Quantitative, qualitative, neuropsychological, and biological, vol. 2. United States: American Psychological Association; 2012. p. 57–71.

[24] Braun V, Clarke V (2019). Reflecting on reflexive thematic analysis. Qual Res Sport Exerc Health.

[25] Evans N, Meñaca A, Andrew EV (2012). Systematic review of the primary research on minority ethnic groups and end-of-life care from the United Kingdom. J Pain Symptom Manage.

[26] Malterud K, Siersma VD, Guassora AD. Sample size in qualitative interview studies: guided by information power. 2016;26(13):1753-60. Available from: https://journals.sagepub.com/doi/abs/10.1177/1049732315617444. 10.1177/1049732315617444 26613970

[27] Teddlie C, Yu F (2007). Mixed methods sampling: a typology with examples. J Mixed Methods Res.

[28] Morrison J, Choudhary C, Beazley R (2023). Observational study of survival outcomes of people referred for ‘fast-track’ end-of-life care funding in a district general hospital: too little too late?. BMJ Open Qual.

[29] Weetman K, Dale J, Spencer R, et al. GP perspectives on hospital discharge letters: an interview and focus group study. Br J Gen Pract Open. 2020. Available from: https://bjgpopen.org/content/4/2/bjgpopen20X101031. Accessed 10/06/20. 10.3399/bjgpopen20X101031 PMC7330207 32398346

[30] NHS Health Research Authority (HRA). Informing participants and seeking consent. Last updated on 4 Sep 2019 ed. 2019. Available from https://www.hra.nhs.uk/planning-and-improving-research/best-practice/informing-participants-and-seeking-consent/.

[31] Parsons JE, Dale J, MacArtney JI (2021). Caring for each other: a rapid review of how mutual dependency is challenged by advanced illness. Int J Care Caring.

[32] Braun V, Clarke V, Hayfield N, Liamputtong P (2019). Thematic analysis. Handbook of research methods in health social sciences.

[33] Braun V, Clarke V (2006). Using thematic analysis in psychology. Qual Res Psychol.

[34] Manera K, Hanson CS, Gutman T, Liamputtong P (2019). Consensus methods: nominal group technique. Handbook of research methods in health social sciences.

[35] Wladkowski SP, Wallace CL. The Forgotten and Misdiagnosed Care Transition: Live Discharge From Hospice Care. Gerontol Geriatr Med. 2022;8:23337214221109984. 10.1177/23337214221109984 PMC9280841 35846976

[36] Professional Records Standards Body. eDischarge summary standard. 2020. Available from: https://theprsb.org/standards/edischargesummary/. Accessed 24 June 2020.

