# Supplementary material for: Improving patients’, carers’ and primary care healthcare professionals’ experiences of discharge communication from specialist palliative care to community settings: a protocol for a qualitative interview study
Source: BMC Palliat Care. 2024 Jun 20;23:156. doi: 10.1186/s12904-024-01451-1 (PMC11191294; doi:10.1186/s12904-024-01451-1)
Supplement: Supplementary file 1 — Supplementary Material 1. [file 12904_2024_1451_MOESM1_ESM.pdf]

Institute of Clinical Sciences, Birmingham Medical School, University of Birmingham
[redacted]

Supplementary file 1 Patient and carer interview schedule

I: Interviewer (member of the research team) *Action points Q= Question

I: Hello my name is X. I am a researcher from University of Birmingham / Warwick and a member of a team that are looking to hear about people's experiences of being discharged form specialist palliative care services. This study has a focus on improving communication.

Please take this time to re-read and familiarise yourself with the materials you received in advance to include the invitation letter, consent form and participant information sheet.

Do you have any questions? **run through forms details and answer queries as required**

Please could you summarise for me what taking part in this research involves?

*if participant(s) understands project involvement *continue*, if not run through information together and repeat question*

If you are happy to proceed with the interview, we need to complete the consent form and we will begin. This is your choice and you may leave without taking part now or at any time.

For joint interviews only: If one or the other of you decide not to take part, the other may continue with the interview, if you wish.

form signing/checking/completion

Note to researcher to separately record consent verbally for applicable proxy signature cases.

This interview will be semi-formal. This means that while I do have some planned questions to ask you, *I want to hear about anything that you say is relevant to your experience of being discharged from specialist palliative care (hospital or hospice)*.

Confirm verbally: "the interview will be recorded and active participation indicates consent to this recording and for your data to be used as outlined in the participant information leaflet. *You may choose not to participate or to end the interview if you do not consent to being recorded or use of your data. The recording will now begin"* ***<u>*START RECORDING*</u>***

My question to begin today is:

Q1: Please tell me about your (or the person you are caring for) experiences of being discharged from specialist palliative care service [name service, if known]?

Potential follow-ups and prompts (not all will necessarily be used or relevant for every interview)

Experience of discharge from specialist palliative care

Q: Can you tell me about how you or the person you are caring for came to be with [specialist palliative care service] and what they have been supporting you/the person you are caring for with?

Q. Can you tell me what your understanding is of why you or the person you are caring for were discharged from specialist palliative care? Are you happy with this decision?

[redacted]

Discharge communication from palliative care

Q: What information were you (patient or carer) given – verbally or in writing – when you were discharged from palliative care [if possible, name specialist palliative care service]? What is your preferred form for this information?

Q. Do you know if any information about your discharge was also given to the person you are caring for or your primary carer (partner/ family member/ close person)? If so, can you say what this was and if it was helpful?

Q: Do you have a copy of the discharge letter? If so, how did you feel about what was written in it?

Q: Would you prefer to receive or not receive palliative care [discharge] letters sent between your healthcare professionals?

Q: How did you feel about the communication during your discharge/care transition?

Preferred place and providers of (palliative) care

Q. How do you feel about the health and social care services that are supporting you currently? [Prompts include: GP, District Nurse, Specialist Palliative Community Nursing Service, Community Palliative Care Doctor, Hospice-At-Home, Social Workers, Night Services, Support Workers].

Q. What are your current preferences for how you or the person you are caring for are supported currently? Is there any support you would like, but are not getting?

Discharge communication preferences

Q: What other information would you have liked to have been given or sent when being discharged from your hospital/hospice [name specialist palliative care service]?

Q: Would you prefer to receive a direct copy of the letter sent to your GP or a separate letter specifically addressed to yourself (or both)?

Q: How do you think the process of patients receiving written discharge communication can be improved [as a carer]?

Q: What information would you like to be given when being discharged and why?

Closing

Q: How do you think the process of palliative care discharge communication can be improved?

Q: Is there anything else you would like to talk to me about today related to this study?

**Discussion may continue in a relaxed conversational manner and researcher may ask additional questions related to anything else relevant mentioned by the patient*.*

Post interview

- Inform participants that the interview is now finished & stop recording
- Ask them how they found taking part & invite them to ask further questions
- Briefly remind participants what the interview will contribute to
- Thank them for their time
