# Supplementary material for: Improving patients’, carers’ and primary care healthcare professionals’ experiences of discharge communication from specialist palliative care to community settings: a protocol for a qualitative interview study
Source: BMC Palliat Care. 2024 Jun 20;23:156. doi: 10.1186/s12904-024-01451-1 (PMC11191294; doi:10.1186/s12904-024-01451-1)
Supplement: Supplementary file 3 — Supplementary Material 3. [file 12904_2024_1451_MOESM3_ESM.pdf]

Centre/Site:

Improving patients', carers' and primary care healthcare professionals' experiences of discharge communication from specialist palliative care to community settings: a qualitative interview study

Supplementary file 3 Study consent form

Participant Consent form

Please tick boxes with black pen or, if using an electronic device, click boxes to "tick"

1. I confirm that I have read the information sheet dated................... (version......) for the above study. I have had the opportunity to consider the information, ask questions and have had these answered satisfactorily.	☐
2. I understand that my participation is voluntary and that I am free to withdraw at any time without giving any reason, without my medical care being affected or (for healthcare professionals only) my employment rights being affected.	☐
3. I understand that data collected during the study, may be shared and/or looked at by authorised individuals from the University of Birmingham and University of Warwick, or from regulatory authorities, where it is relevant to my taking part.	☐
4. I understand that the information collected about me may be used to support other research in the future, and may be shared with other researchers anonymously.	☐
5. I understand that the information collected about me may be shared anonymously (e.g. research publication), without use of my name or anything that identifies me. I understand this may include direct quotations.	☐
6. I agree to be audio and/or video recorded for the study interview.	☐
7. I agree to take part in this study	☐

UNIVERSITY OF BIRMINGHAM | COLLEGE OF MEDICAL AND DENTAL SCIENCES

______________________________ ______________ ______________________________

Name of participant **Date** **Signature of participant/proxy**

If relevant - Name and role (e.g. partner/carer) of proxy ____________________________

________________________________ ___________ _____________________

Name of person seeking consent **Date** **Signature**

If you would like to hear about the study results, please provide your contact details:

____

Study Lead Investigators and contact details:

Dr Katharine Weetman, University of Birmingham, Email: k.e.weetman@bham.ac.uk
Dr John MacArtney, University of Warwick, Email: John.Macartney@warwick.ac.uk

When completed: 1 for participant (optional); 1 for researcher site file.

UNIVERSITY OF BIRMINGHAM | Edgbaston, Birmingham, B15 2TT, United Kingdom www.birmingham.ac.uk
WARWICK THE UNIVERSITY OF WARWICK
FUNDED BY NIHR | National Institute for Health and Care Research
